# Norovirus and Sapovirus Epidemiology and Strain Characteristics among Navajo and Apache Infants

**DOI:** 10.1371/journal.pone.0169491

**Published:** 2017-01-03

**Authors:** Lindsay R. Grant, Katherine L. O’Brien, Robert C. Weatherholtz, Raymond Reid, Novalene Goklish, Mathuram Santosham, Umesh Parashar, Jan Vinjé

**Affiliations:** 1 Center for American Indian Health, Johns Hopkins Bloomberg School of Public Health, Baltimore, Maryland, United States of America; 2 National Center for Immunization and Respiratory Diseases, Centers for Disease Control and Prevention, Atlanta, Georgia, United States of America; University of Michigan, USA, UNITED STATES

## Abstract

Norovirus and sapovirus are important causes of acute gastroenteritis (AGE) among American Indian infants. We investigated the prevalence and molecular epidemiology of norovirus and sapovirus in American Indian infants who have historically experienced a high burden of AGE compared to other US populations. Stool samples were collected from 241 children with AGE (cases) and from 343 infants without AGE (controls) ≤9 months of age from 2002–2004. Cases experienced forceful vomiting and/or 3 or more watery or looser-than-normal stools in 24 hours. Stools were tested by real-time RT-PCR for norovirus GI, GII and GIV and sapovirus GI, GII, GIV and GV. Positive samples were genotyped after sequencing conventional RT-PCR products. Norovirus was identified in 76 (31.5%) of the cases and 70 (20.4%) of the controls (p<0.001). GII.3 and GII.4 Farmington Hills were the most frequently identified genotypes in 14.5% and 30.3% of cases and 17.1% and 27.1% of controls, respectively. Sapovirus GI and GII genotypes were identified in 8 (3.3%) of cases and 8 (2.3%) of controls and a single GIV virus was detected in a control. The same norovirus and sapovirus genotypes were circulating in the general U.S. population in the same time period. The high detection rate of norovirus in healthy controls suggests significant asymptomatic transmission in young infants in these communities.

## Introduction

Norovirus and sapovirus are genetically diverse genera in the family *Caliciviridae*. The major structural capsid protein (VP1) sequence is used to classify both norovirus and sapovirus into genogroups and genotypes. Norovirus is organized into at least seven genogroups (GI-GVII) of which GI, GII and GIV viruses are detected in humans [1]. Of the eight reported sapovirus genogroups (GI-GVIII), viruses of four (GI, GII, GIV and GV) have been detected in humans [2].

In the United States (US), norovirus causes an estimated 19–21 million cases of acute gastroenteritis (AGE) resulting in 1.7–1.9 million outpatient and 400,000 emergency department visits annually [3]. In addition, norovirus causes 56,000–71,000 hospitalizations and 570–800 deaths per year in the US, the majority occurring among young children and the elderly [3]. With the application of real-time RT-PCR diagnostics, sapovirus has been increasingly implicated as the cause of outbreaks [4, 5] and sporadic AGE [6–8]. In pediatric AGE, sapovirus detection ranges from 5–17% depending upon the country [6–10].

Norovirus and sapovirus are important causes of AGE among American Indian infants [11] who have historically experienced a higher burden of AGE compared to children of the general US population [12]. The aim of this analysis is to establish the prevalence and molecular characteristics of the norovirus and sapovirus genotypes circulating among American Indian infants who were selected for a case-control study of AGE etiology [11]. The infants selected for the case-control study were participants of the placebo arm of a rotavirus vaccine efficacy trial that was conducted in two American Indian communities in the Southwest US from 2002–2004 [13].

## Materials and Methods

### The study

Stool samples from American Indian infants ≤9 months of age that had been stored at -80°C since the efficacy trial were selected for the case-control study and tested for adenovirus group F, astrovirus types 1–8, norovirus GI, GII and GIV, group A rotavirus and sapovirus GI, GII, GIV and GV [11]. Case and control inclusion criteria were described previously [11].

### Detection and genotyping of norovirus and sapovirus by real-time RT-PCR

Detection of noroviruses and sapoviruses by real-time RT-PCR has been described in detail elsewhere [11]. Positive specimens were genotyped by conventional RT-PCR [14, 15] and PCR products of appropriate size (norovirus GI: 330 base pairs, norovirus GII: 344 base pairs, sapovirus: 434 base pairs) were gel-purified using the QIAquick Gel Extraction Kit (Qiagen). Sanger sequencing was performed using the Big Dye Cycle Sequencing Kit (Applied Biosystems) and sequences (available upon request) were genotyped by comparison to norovirus and sapovirus reference strains in the database of the National Calicivirus laboratory at CDC. Phylogenetic analyses were performed using MEGA (version 6.0) and statistical analyses were performed in Stata (version 13).

The Institutional Review Boards of the Navajo Nation, Phoenix Area Indian Health Service, Centers for Disease Control and Prevention and Johns Hopkins Bloomberg School of Public Health approved this research, as did the Navajo and White Mountain Apache tribes. Parents provided written consented for participation of their infants in the original rotavirus vaccine trial.

### Results

Norovirus or sapovirus was detected in 84 (35%) of 241 cases and in 79 (23%) of 343 controls. Co-infections of norovirus or sapovirus with another virus were common (Table 1).

**Table 1:** Percent of enteric virus co-infections with norovirus or sapovirus.

Co-infecting virus	Norovirus, N (%)		Sapovirus, N (%)	
	Case (N = 76)	Control* (N = 70)	Case (N = 8)	Control* (N = 9)
Astrovirus	7 (9.2)	7 (10.0)	0 (0)	2 (22.2)
Adenovirus	3 (3.9)	3 (4.3)	2 (25.0)	0 (0)
Rotavirus	8 (10.5)	9 (12.9)	3 (37.5)	2 (22.2)

* There was one control co-infected with norovirus and sapovirus.

Norovirus GII viruses were detected in 71 (29.5%) of cases and in 62 (18.1%) of controls (p<0.001; Table 2). Between 2–3% of cases and controls were positive for norovirus GI.

**Table 2:** Norovirus and sapovirus detected in cases and controls.

Virus	Cases, N = 241		Controls, N = 343	
	N (%)*	N not mixed (%)	N (%)*	N not mixed (%)
Norovirus	76 (31.5)**	59 (24.5)**	70 (20.4)	50 (14.6)
GI	6 (2.5)	6 (2.5)	10 (2.9)	7 (2.0)
GII	71 (29.5)**	54 (22.4)**	62 (18.1)	44 (12.8)
Sapovirus	8 (3.3)	4 (1.7)	9 (2.6)	4 (1.2)

* Includes some cases or controls with mixed viral detection of Group F adenovirus, astrovirus or rotavirus.

**Proportion of cases positive statistically higher than controls (p<0.001).

Norovirus genotypes GII.3 and GII.4 predominated among cases (GII.3: N = 11 [14.5%], GII.4: N = 23 [30.3%]) as well as among controls (GII.3: N = 12 [17.1%], GII.4: N = 19 [27.1%]). Among GI viruses, GI.3b and GI.7 were most prevalent (Table 3).

**Table 3:** Norovirus genotype distribution among cases and healthy controls.

Genotypes	Frequency (%)	
	Cases, N = 76	Controls, N = 70
GI genotypes		
GI.3b	3 (4.0)	1 (1.4)
GI.7	3 (4.0)	6 (8.6)
Not typed	—	3 (4.3)
GII genotypes		
GII.2	1 (1.3)	1 (1.4)
GII.3	11 (14.5)	12 (17.1)
GII.4 Farmington Hills	23 (30.3)	19 (27.1)
GII.5	8 (10.5)	9 (12.9)
GII.6	7 (9.2)	1 (1.4)
GII.7	5 (6.6)	5 (7.1)
GII.12	1 (1.3)	1 (1.4)
GII.14	—	1 (1.4)
GII.17	1 (1.3)	—
Not typed	14 (18.4)	13 (18.6)

Most sapovirus strains belonged to GI (53%) or GII (41%) whereas one GIV virus was detected in a control and no GV viruses were detected (Table 4).

**Table 4:** Sapovirus genotype distribution among cases and healthy controls.

Genotypes	Frequency (%)	
	Cases, N = 8	Controls, N = 9
GI genotypes		
GI.1*	6 (75)	2 (22.2)
GI.2	—	1 (11.1)
GII genotypes		
GII.1*	2 (25)	2 (22.2)
GII.2	2 (25)	1 (11.1)
GIV	—	1 (11.1)
Not typed	1 (12.5)	4 (44.4)

* Both GI.1 and GII.1 viruses were detected in samples from two cases and two controls

## Discussion

We report the prevalence and genotype distribution of norovirus and sapovirus in American Indian infants of the US Southwest from 2002–2004. The prevalence of norovirus in cases (31.5%) was approximately twice as high compared to the estimated global prevalence of 18% [16]. The high prevalence of norovirus found in healthy controls (20.4%) is within the range (4–27%) observed in other pediatric AGE studies [17, 18–21]. Prolonged viral shedding or an altered gut microbiota have been suggested as possible explanations of high detection rates among control subjects [22, 23].

Norovirus GII.3 and GII.4 were the primary genotypes identified among American Indian infants. These genotypes have been reported globally but also vary geographically [17, 24–26]. Several studies from Africa suggested a higher prevalence of GII.3 viruses in children [24–26], but not in another study in young children the U.S. [17]. All GII.4 viruses detected in the American Indian children typed as GII.4 Farmington Hills, a variant that emerged in 2002 [27] and became the predominant strain globally [28].

This study had several limitations. The stools from this study were collected 11–13 years ago in the context of a clinical trial for a rotavirus vaccine candidate. Therefore, the characterized genotypes may not reflect the current distribution of norovirus and sapovirus circulating among American Indian infants in the US Southwest. The high prevalence of norovirus and sapovirus in healthy controls creates difficulty in the attribution of AGE etiology and dampens the association between virus detection and disease and future studies should focus on whether prolonged shedding could explain the high percentage of positives in healthy control children.

Norovirus GII.3 and GII.4 genotypes predominated among Navajo and White Mountain Apache infants in the early 2000’s as well as in norovirus outbreaks and among other populations in the U.S. around that same time [27, 29]. A similar distribution of norovirus genotypes among cases and controls suggest significant transmission within the population accompanied by asymptomatic carriage and shedding of the virus. Only a few different sapovirus genotypes were detected among American Indian infants. The frequent number of co-infections in children highlights that continued monitoring of the causes of AGE should include multi-enteric diagnostic platforms [30] to advance our understanding of the individual AGE pathogen in high-risk populations. It would be beneficial to conduct another study in this population to determine if norovirus trends and strain characteristics have changed over time.
